# *ArrayIDer*: automated structural re-annotation pipeline for DNA microarrays

**DOI:** 10.1186/1471-2105-10-30

**Published:** 2009-01-23

**Authors:** Bart HJ van den Berg, Jay H Konieczka, Fiona M McCarthy, Shane C Burgess

**Affiliations:** 1 Department of Basic Science, PO Box 6100, College of Veterinary Medicine, Mississippi State University, Mississippi State, MS 39762, USA; 2 Instistute for Digital Biology, Mississippi State University, Mississippi State, MS 39762, USA; 3 Department of Molecular & Cellular Biology, University of Arizona, 1656 E. Mabel, MRB 317, Tucson, AZ 85724, USA; 4 Life Sciences and Biotechnology Institute, Mississippi State University, Mississippi State, Mississippi 39762, USA

## Abstract

**Background:**

Systems biology modeling from microarray data requires the most contemporary structural and functional array annotation. However, microarray annotations, especially for non-commercial, non-traditional biomedical model organisms, are often dated. In addition, most microarray analysis tools do not readily accept EST clone names, which are abundantly represented on arrays. Manual re-annotation of microarrays is impracticable and so we developed a computational re-annotation tool (*ArrayIDer*) to retrieve the most recent accession mapping files from public databases based on EST clone names or accessions and rapidly generate database accessions for entire microarrays.

**Results:**

We utilized the Fred Hutchinson Cancer Research Centre 13K chicken cDNA array – a widely-used non-commercial chicken microarray – to demonstrate the principle that *ArrayIDer *could markedly improve annotation. We structurally re-annotated 55% of the entire array. Moreover, we decreased non-chicken functional annotations by 2 fold. One beneficial consequence of our re-annotation was to identify 290 pseudogenes, of which 66 were previously incorrectly annotated.

**Conclusion:**

*ArrayIDer *allows rapid automated structural re-annotation of entire arrays and provides multiple accession types for use in subsequent functional analysis. This information is especially valuable for systems biology modeling in the non-traditional biomedical model organisms.

## Background

Microarrays have become a standard tool for functional genomics allowing analysis of thousands of mRNA transcripts simultaneously and they are widely used for a diverse range of species [1-4]. Microarrays have been applied to species regardless of whether or not their whole genome sequence is available. However, understanding the biological meaning represented by microarray data is hindered by lack of structural – and functional annotation (i.e. identifying the genes represented on arrays and linking these to functional information, respectively). Despite the prevalence of EST sequences represented on microarrays for most species, existing tools for expression data analyses and array functional annotation [5-11] do not accept EST clone names or accessions as input. Therefore, researchers are often hindered to first convert EST clone names or accession numbers to identifiers compatible with these functional analyses tools.

Although 10 software packages have been developed to map between popular database identifiers [5,9,10,12-18], these gene cross-reference tools are not compatible with EST clone name input, focus only on widely-used commercially-available arrays or only incorporate limited organisms. Moreover, functional information (such as the Gene Ontology) is associated with National Center for Biotechnology Information (NCBI) and UniProtKB accessions and annotations provided by vendors, or researchers who provide non-commercial arrays, may be very dated. Although *EasyGO *[18] annotates several traditionally-agricultural species' microarrays, users cannot directly access the assigned structural annotations for downstream functional analysis. To derive value from microarray experiments, especially as research in more species becomes enabled by microarray technology, it is crucial to improve the annotation for existing arrays in ways that is accessible for array users.

Here we describe *ArrayIDer*, a user-friendly program that generates a library of public accessions available from the Gene Expression Omnibus (GEO) browser [19] for both custom-made and commercial arrays. *ArrayIDer *currently accepts data from any microarray containing EST identifiers compatible with the NCBI UniGene database [20] from nine species: human, mouse, rat, horse, pig, chicken, cow, Arabidopsis, and zebra fish. The program accepts either cDNA/EST clone names or the corresponding GenBank EST nucleotide accession. *ArrayIDer *generates a library of gene and protein accessions from the latest updated NCBI UniGene [20] and International Protein Index (IPI) [21] databases. *ArrayIDer *retrieves identifiers from UniGene and IPI that match the EST input list. All annotations of ESTs to genes (and accompanied proteins) are as assigned by NCBI UniGene. ESTs listed in UniGene are grouped in a UniGene cluster based on their nucleotide overlap. The gene represented by each cluster is determined by the top BlastX hit of the nucleotide sequence. Gene information regarding the EST cluster to gene match is retrieved from the NCBI Homologene database, where known orthologs for genes are mapped through multiple species. The structural annotations retrieved by *ArrayIDer *are only retrieved from the species-specific UniGene database, which contains pre-assigned structural annotations made according to the methods used at the Homologene database. An online version of *ArrayIDer *allows rapid identifier searching of EST libraries of several species generated by *AgBase*.

## Implementation

Microarray libraries for multiple species generated with *ArrayIDer *are available at the *AgBase *website  → Array Annotation → *ArrayIDer*) and researchers can use the simple interface to search structural annotations for their microarray ESTs or accessions in the species' EST library. Libraries available online are updated when new versions of the underlying databases are released. Any available library can be extended by users by contacting *AgBase *directly to request structural annotation for their arrays. Conversely, and especially for those conversant with Perl, *ArrayIDer *is available for download for researchers to generate a library for species currently not listed on *AgBase *to avoid requesting the work be done by *AgBase *staff. *ArrayIDer *runs locally via the command line console or by execution in a designated directory. To run locally *ArrayIDer *requires: 1) Perl platform (version 5.8.8 built 8.17 or higher); 2) installation of Archive::Extract, DBI and NET::FTP Perl modules; 3) a text formatted input file of cDNA/EST clone names or GenBank nucleotide sequence accessions; and 4) an internet connection. The script downloads and unpacks the required databases directly from the internet.

**Figure 1:**
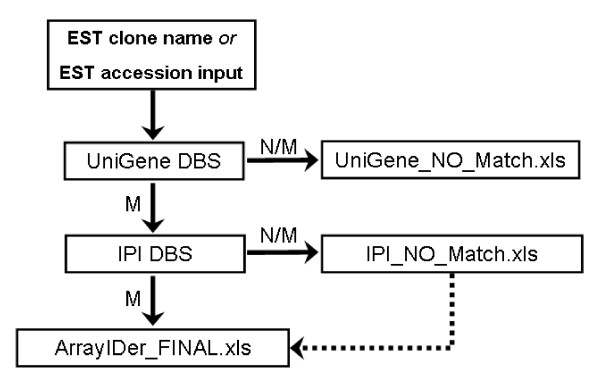
***ArrayIDer *output structure**. EST clone names or EST accession numbers from input are searched against the UniGene database (DBS). Accessions without match (N/M = no match) are written to UniGene_NO_Match.xls. Matching accessions (M = Match) are searched against the IPI database. Accessions with no IPI matches are written to IPI_NO_Match.xls. Matching accessions are written to ArrayIDer_FINAL.xls. All data from IPI_NO_Match.xls is also written to ArrayIDer_FINAL.xls, since it contains identifier information retrieved from the UniGene database.

The *ArrayIDer *standalone version reads the input list and searches each entry against the latest version of NCBI UniGene to retrieve initial gene and protein information (Figure Fig. 1). Input entries with no UniGene match are written to an output file (UniGene_NO_MATCH.xls). Input entries that match a UniGene ID are used to search the IPI database for additional gene and protein identifiers information. Input entries with no IPI match are written to a second output file (IPI_NO_MATCH.xls). Input entries with IPI match are written to a third output file (ArrayIDer_FINAL.xls) containing accessions matching both UniGene and IPI and accessions with only UniGene matches. ArrayIDer_FINAL.xls contains 13 different types of accessions from 7 public databases (Table 1).

**Table 1:** *ArrayIDer *output description.

Column Name	Type of information
**CLONE_NAME**	**EST clone name from array**

**NUCL_GB_ACC**	**GenBank nucleotide accession from clone sequence**

SEQ_TYPE	mRNA or EST

UNIGENE_ID	Corresponding NCBI UniGene database identifier

GENE_SYMBOL	Gene symbol provided by NCBI

GENE_ID	NCBI Entrez gene identifier

PROT_GB_ACC	Corresponding NCBI Protein accession number(s)

PROT_GI_NO	Corresponding NCBI Protein GI number(s)

PEPT_ACC	Corresponding Peptide accession(s)

Retrieval DB	Database of additional retrieved protein accession (Swiss-Prot/TrEMBL; RefSEQ, ENSMBLE)

DB_ACC	Accession number(s) corresponding to Retrieval DB

IPI_ID	Corresponding IPI identifier(s)

UNIPROT_ACC	Corresponding UniProtKB accession(s)

ENSEMBL_ID	Corresponding ENSEMBL identifier(s)

UNIPARC_ID	Corresponding UniParc identifier(s)

The input information is indicated in bold text and the different types of information retrieved by *ArrayIDer *are shown. Identifiers can be used to cross-reference several other publicly available databases to retrieve additional information for genes of interest.

**Figure 2:**
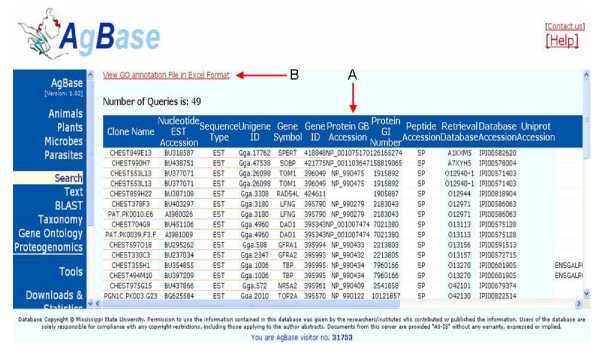
**Result format for ArrayIDer online**. (A) The table contains a list with all input matches to the NCBI UniGene and IPI database. The results can be exported in Excel format using the link (B).

The online *ArrayIDer *library for a species contains the same table as the standalone version ArrayIDer_FINAL.xls output file for the same species. However, the online libraries also contain additional mapped EST or clone name identifiers assigned by *AgBase *using additional methods (including functional domain mapping, manual database searching and identifier correction) for comprehensive identifier mapping. An example of the online output can be found in Figure Fig. 2.

## Results and discussion

*ArrayIDer *allows researchers to rapidly update the structural annotation of their microarray and use this information in downstream gene expression modeling and pathways analysis. To demonstrate the use of *ArrayIDer *we selected a widely-used non-commercial array, the Fred Hutchinson Cancer Research Centre (FHCRC) 13K chicken cDNA array [22]. The array's data table was downloaded from the NCBI GEO browser (accession GPL2863).

### Data Output and performance

Microarray structural re-annotation results were compared to the annotation currently provided for the FHCRC array. In total, 13,234 probe identifiers were submitted as input for the script. Originally, 1136 array probes were structurally annotated to a chicken gene, with a further 7820 structural annotations to other (non-chicken) species (including fruit fly, sea snake, and frog). *ArrayIDer *provided a 6.67-fold increase (1,136 vs. 7,581) in chicken-specific annotations. Among the chicken structural annotations assigned, 55% (4177) are assigned to a Swiss-Prot/TrEMBL accession. 45% (3404) are assigned to a predicted "XP_" accession, which are candidates for further annotation curation. Identification and curation of these XP_ accessions improves the species' genome annotation.

### Identification of Pseudogenes

Initially, we identified 290 transcripts on the FHCRC array with *ArrayIDer *that mapped to gene elements labelled as pseudogenes. Pseudogenes have been defined as defunct relatives of known genes that are considered non-functional; however, some pseudogenes can be transcribed and play a role in gene regulation and expression [23]. Moreover, pseudogenes are difficult to identify and may be miss-annotated by genome annotation. Manual inspection of the identified pseudogenes from this array (including BLAST analysis and synteny) found 66 gene elements that are likely to be functional. We have submitted these changes to NCBI and *AgBase*. Four months after submission of the changes, we re-analyzed the FHCRC array with *ArrayIDer *and identified the remaining 224 gene elements labelled as pseudogenes, indicating the 66 genes are corrected and updated in the public database.

### Improved Functional annotation

The Gene Ontology (GO) is the de facto standard method for functional annotation of gene products [24]. While ESTs are represented on arrays, GO annotation is linked to gene or protein IDs. By linking ESTs represented on arrays to protein or gene ac-cessions, we can associate function to array elements. For example, using the FHCRC chicken cDNA array, chicken-specific GO annotations are highly desired. If no chicken-specific annotations are available, non-chicken annotations can be used for deriving biological value from the data. Using *ArrayIDer*, we were able to decrease non-chicken functional annotations associated with the array by 2 fold (from 7309 to 3671).

## Conclusion

Continual structural- and functional re-annotation of microarrays ensures the most up-to-date gene product information for modeling functional genomics datasets. *ArrayIDer *allows rapid automated re-annotation of entire arrays and provides the user with multiple accession types for use in functional analysis. Together this information is especially valuable for the non-traditional biomedical model organisms to utilize the wide range of existing tools for systems biology modeling downstream. We focus on expanding the number of public databases used to assign accessions, include up-to-date, curated functional annotations for both commercial and custom designed microarrays (including specific requests) and incorporate this information into the *AgBase *database for user-friendly online access.

## Availability and requirements

Project name: *ArrayIDer*

Project home page:  → Array annotation → *ArrayIDer*

Operating system(s): Platform independent

Programming language: Perl

Other requirements: Perl modules Archive::Extract, DBI and NET::FTP

License: Freely available

## Authors' contributions

BVDB developed the pipeline, compiled the tool's script and drafted the manuscript. JHK contributed in the tool's script optimization and performance evaluation. FMM and SCB participated in the pipeline development and help draft the manuscript. All authors read and approved the final manuscript.
